# Characterization and Antimicrobial Property of Poly(Acrylic Acid) Nanogel Containing Silver Particle Prepared by Electron Beam

**DOI:** 10.3390/ijms140611011

**Published:** 2013-05-24

**Authors:** Jong-Bae Choi, Jong-Seok Park, Myung-Seob Khil, Hui-Jeong Gwon, Youn-Mook Lim, Sung-In Jeong, Young-Min Shin, Young-Chang Nho

**Affiliations:** 1 Advanced Radiation Technology Institute, Korea Atomic Energy Research Institute, 1266 Sinjeong-dong Jeongeup-si, Jeollabuk-do 580-185, Korea; E-Mails: jbchoi@kaeri.re.kr (J.-B.C.); hjgwon@kaeri.re.kr (H.-J.G.); ymlim71@kaeri.re.kr (Y.-M.L.); sijeong@kaeri.re.kr (S.-I.J.); ymshin@kaeri.re.kr (Y.-M.S.); ycnho@kaeri.re.kr (Y.-C.N.); 2 Department of Organic Materials and Fibers Engineering, Chonbuk National University, Jeonju 561-756, Korea; E-Mail: mskhil@jbnu.ac.kr

**Keywords:** nanogel, poly(acrylic acid), silver nanoparticle, antimicrobial property

## Abstract

In this study, we developed a one step process to synthesize nanogel containing silver nanoparticles involving electron beam irradiation. Water-soluble silver nitrate powder is dissolved in the distilled water and then poly(acrylic acid) (PAAc) and hexane are put into this silver nitrate solution. These samples are irradiated by an electron beam to make the PAAc nanogels containing silver nanoparticles (Ag/PAAc nanogels). The nanoparticles were characterized by scanning electron microscopy (SEM) and energy dispersive spectroscopy (EDS). In addition, the particle size and zeta-potential were confirmed by a particle size analyzer (PSA). The antibacterial properties of the nanogels were evaluated by paper diffusion test. The Ag/PAAc nanogels had an antibacterial effect against *Escherichia coli* and *Staphylococcus aureus*. The nanogels also demonstrated a good healing effect against diabetic ulcer. The size of the Ag/PAAc nanogels decreased with increasing irradiation doses, and the absolute value of the zeta potential increased with increasing irradiation doses. Also, the Ag/PAAc nanogels exhibited good antibacterial activity against both Gram-negative and Gram-positive bacteria. In *in vivo* wound healing, the Ag/PAAc nanogels have a good healing effect.

## 1. Introduction

Nanogels are internally cross-linked particles of sub-micrometer size made of hydrophilic polymers [1] and are considered to be a distinct type of macromolecule, compared to linear and branched polymers or macroscopic gels [2]. Such structures, along with their bigger analogues—microgels—are tested for a number of practical applications, starting from fillers in a coating industry to “smart” drug delivery systems [3].

Nanogels from hydrophilic polymer matrices are crosslinked by several methods. One method is physical crosslinking, such as hydrogen bonds, crystallized domains, hydrophobic interactions and temperature-induced sol-gel transition. These physically crosslinked gels can reversibly degrade into the corresponding precursors upon external stimuli. Another method involves chemical crosslinking in the presence of various crosslinkers. However, the chemical method had a few problems, including toxicity and an elimination of catalysts after a reaction [4]. In this study, radiation technology was used for the nanogel preparation, as this method is capable of sterilization and crosslinking concurrently without a catalyst.

The radiation-induced nanogels can be synthesized using the following two methods: inter-molecular and intra-molecular crosslinking of a linear-chain polymer by radiation [5,6]. The radiation-induced synthesis of nanogels is initiated by irradiation of a dilute aqueous polymer solution. Reactive radical intermediates are formed by this process in the polymer chains. This is the result of indirect effects in that the radiation is absorbed largely by the water to produce several key reactive species, in particular, hydrate electrons (e^−^), hydroxyl radicals (·OH) and hydrogen atoms (H). The hydroxyl radical abstracts and H atom form the polymer chain, producing carbon-centered free radicals along the backbone of the chain. These can then recombine via inter- or intra-molecular crosslinking [7]. As a result, nanogels are produced from intra-molecular recombination of the radicals.

The technique is applied for water-soluble neutral polymers, such as poly(vinyl alcohol), poly(vinyl pyrrolidone), poly(vinyl methyl ether) and an electrolytic polymer, poly(acrylic acid) (PAAc) [8–10]. Specially, PAAc-based polymers are nontoxic and biodegradable, and recently, *in vivo* studies on PAAc-based hydrogels suggest they are well tolerated [11–13].

Silver nanoparticles (Ag NPs) have attracted much attention for centuries due to their unique optical properties, electrical conductivities, oxidative catalysis and antibacterial effect [14]. A series of physical/chemical/photochemical methods were attempted and developed for the synthesis of different size and shape controlled silver particles [15,16], such as lithography, laser ablation, self-assembly technique [16], *etc*. Especially, the use of radiation in the preparation of Ag NPs has been considered as a clean and effective method to reduce Ag^+^ ions at ambient temperature without using excessive reducing agents or producing unwanted by-products [17,18].

However, the existing research method has a drawback: it doesn’t allow one to easily synthesize nanogels containing silver nanoparticles.

The main goal of this study is to develop a one step process to synthesize nanogels containing silver nanoparticles involving electron beam irradiation. In this regard, water-soluble silver nitrate powder is dissolved in distilled water, and then, PAAc and hexane are put into this silver nitrate solution. These samples are irradiated by an electron beam to make the PAAc nanogels containing silver nanoparticles (Ag/PAAc nanogels). These solutions are simultaneously subject to induce intra-molecular crosslinking of PAAc hydrogel and reduce Ag^+^ ions by electron beam irradiation. Characterization of the Ag/PAAc nanogels is carried out, including particle size, morphological structure, antimicrobial property and wound healing for potential application as biomaterials.

## 2. Results and Discussion

### 2.1. Characterization of the Prepared Ag/PAAc Nanogel

In this experiment, the synthesis of Ag/PAAc nanogel simultaneously occurred through reduction of AgNO_3_ and intra-molecular crosslinking of PAAc. The previous work has demonstrated that the UV-Vis absorption spectra are quite sensitive to the formation of Ag NPs; typically, the absorption peaks depend on their particle diameters and shapes. Generally, the UV-Vis absorption spectra are quite sensitive to the formation of Ag NPs; typically, the absorption peaks depend on their particle diameter. Ag NPs have an absorption band in the visible range of 400–500 nm [19].

**Figure 1:**
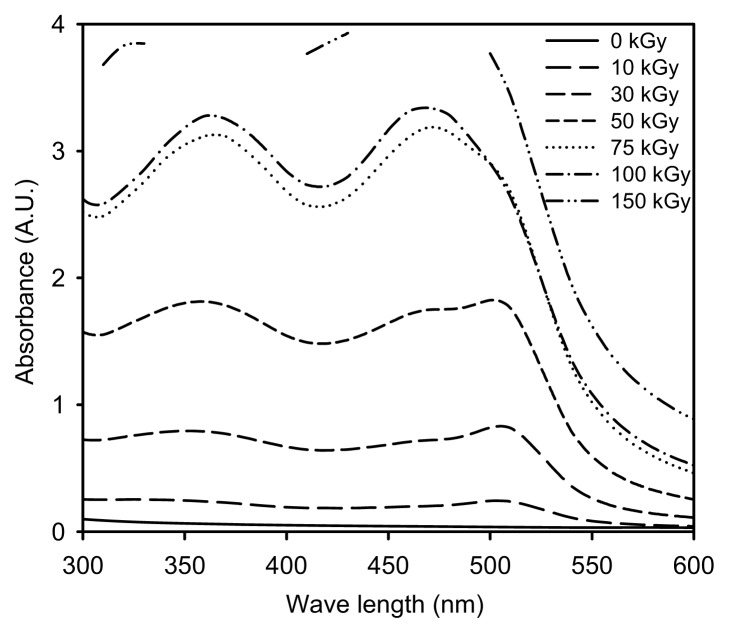
UV-Vis absorption spectra of Ag/PAAc nanogels.

Figure 1 illustrates the absorption spectra for Ag/PAAc nanogels in the range of 300–600 nm. As shown in Figure 1, the intensity of the absorption band of Ag NPs increases significantly and shifts to blue with an increasing absorption dose. The intensity of the absorption band of Ag/PAAc nanogels increased significantly upon increasing the radiation dose, indicating that a correspondingly higher yield of Ag NPs was obtained. In addition, a blue shift of the absorption band of Ag NPs occurred upon the increasing the electron beam irradiation dose. This result suggests that smaller-diameter Ag NPs were obtained at larger absorption doses. In the formation of Ag NPs in the PAAc nanogels, it can be expected that silver nitrate is readily reduced by an electron beam. The aqueous AgNO_3_ solution in PAAc hydrogels was irradiated by an electron beam for creating hydrated electrons, primary radicals and molecules. These solvated electrons and H atoms were strong reducing agents, so this could easily reduce Ag^+^ ions down to the zero-valent state.

This suggestion also could be confirmed by the particle size analyzer (PSA) and FE-SEM micrographs.

**Figure 2:**
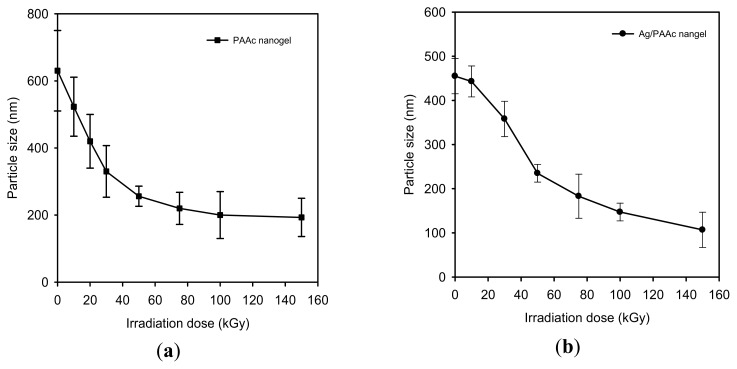
The particle size of (**a**) PAAc nanogel and (**b**) Ag/PAAc nanogel.

**Figure 3:**
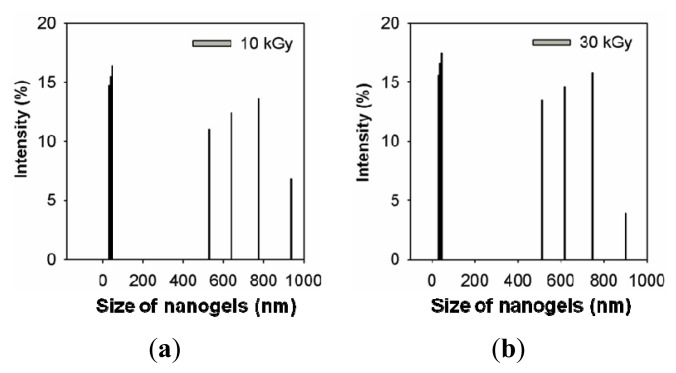

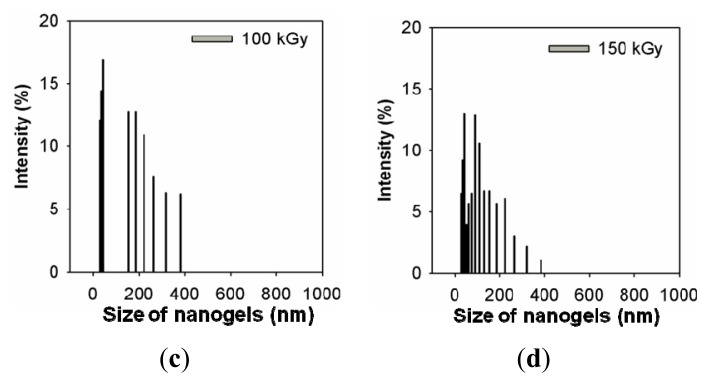
The size distribution of Ag/PAAc nanogels; PAAc 1 wt%, hexane 3 wt% AgNO_3_ 0.005 M (**a**) 10 kGy, (**b**) 30 kGy, (**c**) 100 kGy and (**d**) 150 kGy.

The nanogel size and distribution were measured with a PSA. Figure 2a shows that the sizes of the PAAc nanogels decreased with increasing electron beam irradiation doses. These caused the intramolecular crosslinking of PAAc to occur more at higher irradiation doses than at lower irradiation doses. The average size of the PAAc nanogels was determined to be approximately 200 nm at 100 kGy. This result is based on the interfacial deposition of PAAc after displacement of a polar solvent: water from hexane solution. Rapid diffusion of the hexane solution into the polar solvent phase results in the decrease of interfacial tension between the two phases, which increases the surface area and leads to the formation of small nanogels [20]. Also, the sizes of the Ag NPs/PAAc nanogels (Figure 2b) were regularly decreased with increasing irradiation doses. The use of electron beam irradiation in the preparation of Ag NPs has been considered as the effective method to reduce Ag^+^ ions without using excessive reducing agents. Hence, it is possible to control the size of the Ag/PAAc nanogels by irradiation doses. Figure 3 shows the size distribution of Ag/PAAc nanogels with increasing irradiation doses. As in the PAAc nanogels, the Ag/PAAc nanogels have a smaller size and narrower distribution with an increasing irradiation dose.

**Figure 4:**
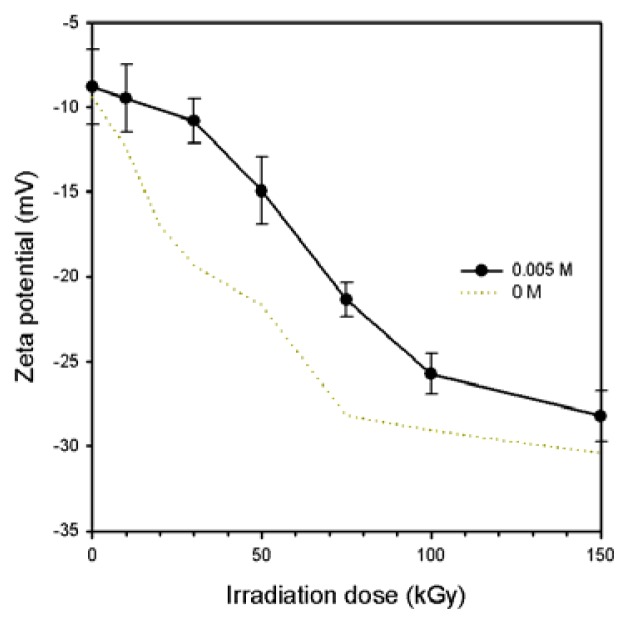
Zeta potential (ζ-potential) of Ag/PAAc nanogels; PAAc 1 wt%, hexane 3 wt%, AgNO_3_ 0.005 M.

A zeta potential is the net surface charge of the nanoparticle when it is inside a solution. The fact that particles push each other and their agglomeration behavior depends on a large negative or positive zeta potential. The zeta potential that plays an important role limits the stability of solutions by +30 mV or −30 mV [21]. Figure 4 shows the zeta potential of the Ag/PAAc nanogels as a function of radiation dose. As shown in Figure 4, the absolute value of the zeta potential was increased with increasing irradiation doses. The highest zeta potential of the Ag/PAAc nanogels was approximately −27 mV at 150 kGy. This means that the Ag/PAAc nanogels at 150 kGy was well distributed and that they are not easily aggregated.

**Figure 5:**
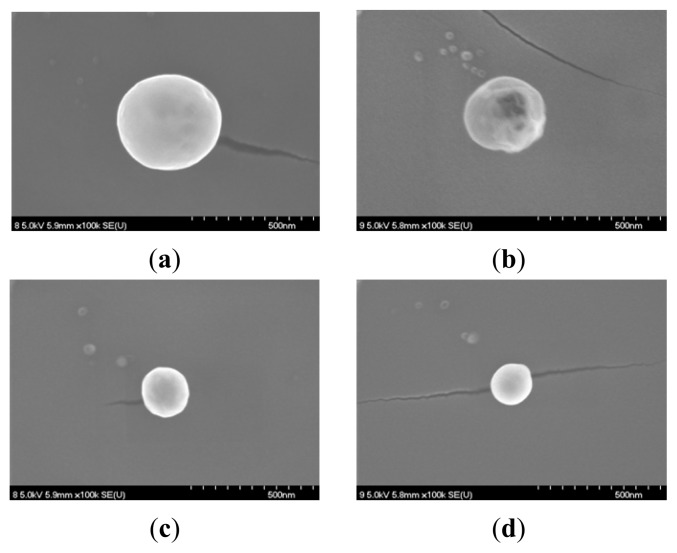
Scanning electron microscopic images of nanogels; PAAc 1 wt%, hexane 3 wt% (**a**) 30 kGy, (**b**) 50 kGy, (**c**) 75 kGy and (**d**) 100 kGy.

**Figure 6:**
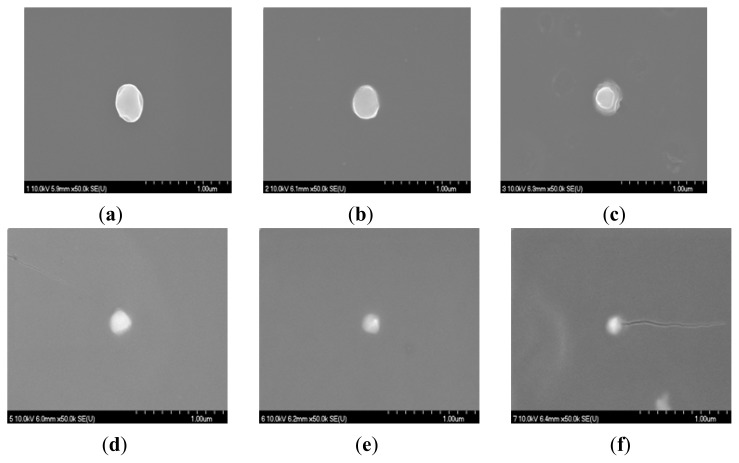
Scanning electron microscopic images of the Ag/PAAc nanogel; PAAc 1 wt%, Hexane 3 wt%, AgNO_3_ 0.005 M (**a**) 0 kGy, (**b**) 10 kGy, (**c**) 30 kGy, (**d**) 75 kGy, (**e**) 100 kGy and (**f**) 150 kGy.

The morphology of the resulting PAAc nanogels and Ag/PAAc nanogels were observed by FE-SEM (Figures 5 and 6). Figure 5 showed the SEM images of the nanogels at PAAc 1 wt%, Hexane 3 wt% with different irradiation doses. It was confirmed from Figure 5 that the PAAc nanogels were spherical in shape, and the size of the nanogels decreased with an increase in irradiation doses. Figure 6 shows the SEM images of the Ag/PAAc nanogels with different irradiation doses. The Ag/PAAc nanogels also were spherical in shape, and the size of the nanogels decreased with an increasing irradiation dose, as in the PAAc nanogels. However, the size of the Ag/PAAc nanogels was smaller than the PAAc nanogels, as in the PSA results, because AgNO_3_ interfered with the PAAc crosslinking.

**Figure 7:**
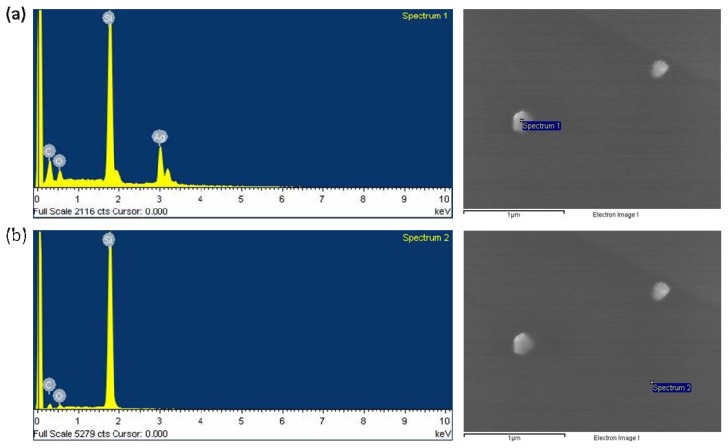
Energy dispersive X-ray spectrum and images of the Ag/PAAc nanogel; PAAc 1 wt%, Hexane 3 wt%, AgNO_3_ 0.005 M, Radiation dose 50 kGy (**a**) particle and (**b**) blank.

To confirm the existence of Ag NPs in the PAAc nanogels, the Ag/PAAc nanogels were dispersed on the silicon wafer substrate. Figure 7a shows the EDX spectrum of the Ag/PAAc nanogels. Ag NPs generally show a typical absorption peak at approximately 3 keV due to the surface plasmon resonance. The presence of the elemental silver can be observed in the graph obtained from the EDX analysis. However, elemental silver was not observed in the blank point (Figure 7b). This indicates the reduction of silver ions into elemental silver. The results of the energy dispersive X-ray spectroscopy analysis also confirmed that silver elements exist in the PAAc nanogels.

### 2.2. Antimicrobial Property of the Prepared Ag/PAAc Nanogel

**Figure 8:**
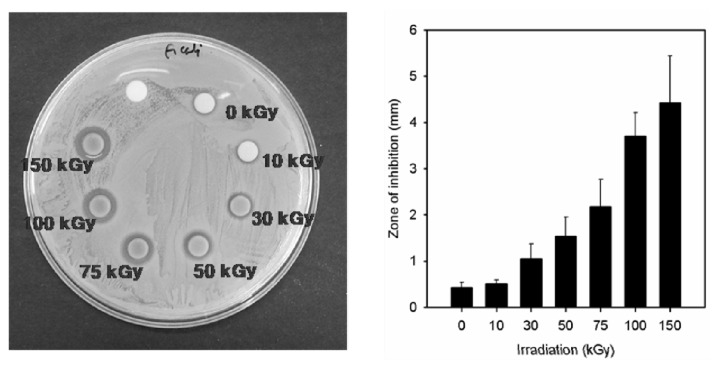
Zone of inhibition of silver nanoparticles against *Escherichia coli*; PAAc 1 wt%, Hexane 3 wt%, AgNO_3_ 0.005 M.

**Figure 9:**
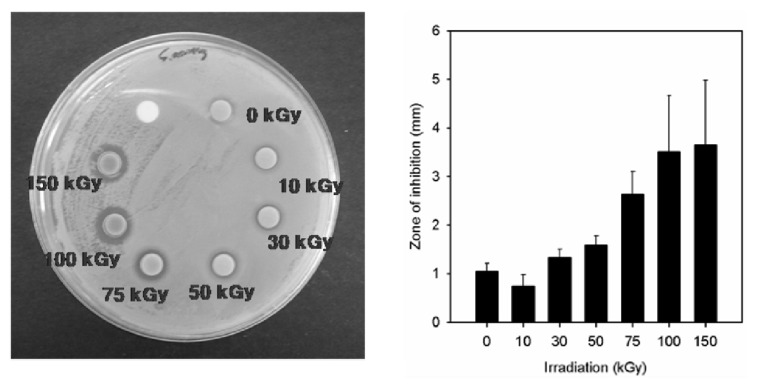
Zone of inhibition of silver nanoparticles against *Staphylococcus aureus*; PAAc 1 wt%, Hexane 3 wt%, AgNO_3_ 0.005 M.

In our study, the antimicrobial properties of Ag NPs synthesized using the PAAc nanogels against *S. aureus* ATCC 6538P and *E. coli* ATCC 25922 have been studied on solid growth media. As shown in Figures 8 and 9, the antibacterial effects of the Ag/PAAc nanogels were increased with an increasing irradiation doses. Also, the Ag/PAAc nanogels exhibited good antibacterial activity against both Gram-negative and Gram-positive bacteria. They showed higher antibacterial activity against *E. coli* than *S. aureus* (Table 1). This result is possibly due to the difference in the structure of the cell wall between the Gram-positive and Gram-negative bacteria. The cell wall of the Gram-positive bacteria is composed of a thick layer of peptidoglycan, consisting of linear polysaccharide chains cross-linked by short peptides, thus forming a more rigid structure leading to a difficult penetration of Ag NPs compared to the Gram-negative bacteria, where the cell wall possesses a thinner layer of peptidoglycan [22]. Also, the antibacterial activity is high in the case of Ag/PAAc nanogels at 150 kGy compared to that at 10 kGy, due to a smaller size of Ag/PAAc nanogels [23,24]. The smaller Ag NPs having a larger surface area for interaction have efficient antibacterial activity compared to the larger Ag NPs. In addition, the high bactericidal activity is certainly due to the silver cations released from Ag NPs that act as reservoirs for the Ag^+^ bactericidal agent. Big changes in the membrane structure of bacteria as a result of the interaction with silver cations lead to the increased membrane permeability of the bacteria [25].

### 2.3. *In Vivo* Wound Healing

**Figure 10:**
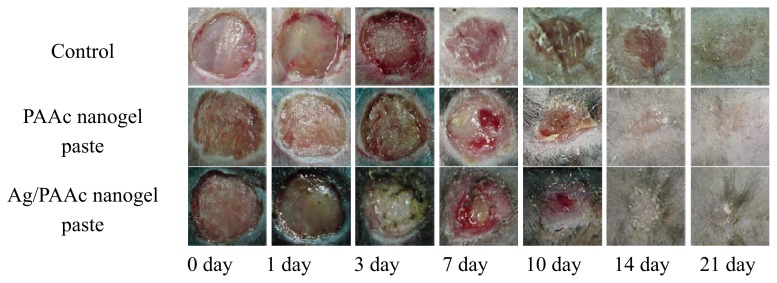
Wound image of the control, commercial medicine, PAAc nanogel paste, Ag/PAAc nanogel paste after one, three, seven, 10, 14, 21 days treated.

The female db/db mice were used for *in vivo* wound healing assessment. Figure 10 shows the wound image and recovery ratio of the ulcer. Three experimental groups, namely, non-treated control wound, the PAAc nanogel paste and the Ag/PAAc nanogel paste, were arranged. The wounds have been observed using a digital camera for three, seven, 10, 14 and 21 days to confirm the recovery ratio of diabetic ulcer. As shown in Figure 10, the amount of wound exudates were observed in all groups until three days, while the wound regeneration was observed after seven days. In the case of the control group, the area of the wound was more spacious than other groups, because the regeneration of the wound did not progress as much. Other groups, the PAAc nanogel paste and the Ag/PAAc nanogel paste, progressed at a similar ratio to the epithelial cell regeneration. Especially, Ag/PAAc nanogel paste hastened the healing rate by accelerating wound contraction compared with wounds treated only with PAAc nanogels. The probable reason for this result is that silver is inactivated by protein and anion complex in ulcer. It is clear in the above stated good reason that silver ions and particles have an excellent antimicrobial activity due to the interfering with the respiratory chain at the cytochromes. Also, silver ions interfere with components of the microbial electron transport system, bind DNA and inhibit DNA replication [26]. These observations reveal that the Ag/PAAc nanogels can meet the efficiency ulcer healing.

## 3. Experimental Section

### 3.1. Materials

Poly(acrylic acid) (PAAc, *M*w = 1.0 × 10^6^) was purchased from Wako chemical Co. (Tokyo, Japan). Hexane and silver nitrate (AgNO_3_) were purchased from Showa chemical Co. (Tokyo, Japan). Experimental deionized (DI) water was produced by a purification system from Young Lin Instrument Co., Ltd. (Seoul, Korea). All chemicals were used without further purification.

### 3.2. Preparation of PAAc Nanogels and Ag/PAAc Nanogels

PAAc nanogels were prepared through electron beam irradiation. One weight percent of PAAc and 3 wt% Hexane were dissolved in distilled water. The solutions were stirred for 18 h at room temperature by an electronic overhead stirrer (MS-DL1020D, MTOPS, Seoul, Korea) at 500 rpm, and the solutions were then poured into square dishes (125 × 125 mm, SPL, Pocheon, Korea). These samples were irradiated by an electron beam accelerator (UELV-10-10S, Moscow, Russia) (beam current of 1 mA and an energy of 10 MeV). The irradiation dose ranged from 10 to 150 kGy. Then, hexane was removed by evaporation.

To prepare the PAAc nanogels containing silver particles (Ag/PAAc nanogels), the silver particles were synthesized from a silver nitrate solution. Water-soluble silver nitrate powder was dissolved in the distilled water, and then PAAc and hexane were put into this silver nitrate solution (0.005 M). The solutions were stirred for 18 h at room temperature by an electronic overhead stirrer (MS-DL1020D, MTOPS,Seoul, Korea) at 500 rpm, and the solutions were then poured into square dishes (125 × 125 mm, SPL, Pocheon, Korea). These samples were irradiated by an electron beam. The irradiation dose ranged from 10 to 150 kGy. Then, hexane was removed by evaporation.

### 3.3. UV-Vis Spectrophotometer

The UV-Vis spectra of the PAAc nanogels and the Ag/PAAc nanogels were recorded using a spectrophotometer (BioTek Instruments, Inc., Winooski, VT, USA) with a range of wavelengths from 200 to 900 nm

### 3.4. Particle Size Distribution and Zeta Potential Analysis

The particle size and zeta potential were measured by photon correlation spectroscopy using a particle size analyzer (Delsa nano C, Beckman Coulter Inc., Winooski, VT, USA). All samples were measured without any chemicals or filtering. The nanogel suspensions were distributed by vortex for 3 min. The nanogel suspensions were then poured into approximately 2 mL quart cells. The sizing measurements were performed at 25 °C.

### 3.5. FE-SEM and EDX Analysis

To observe the high-resolution images of the nanogels, the samples were covered with a layer of osmium (Os) for 60 s by sputter coating. The morphology and size of the nanogels were investigated with a field emission scanning electron microscope (FE-SEM S-4700, Hitachi, Tokyo, Japan) at a resolution of 60 Å at 5 kV, with a magnification of 5.0 K and a working distance of 10–12 mm. The size of nanogels can be calculated using the scale provided in the micrograph.

To confirm the existence of silver particles in the nanogels, the samples were investigated by FE-SEM with energy dispersive X-ray spectrometer (EDX, EMAX, Horiba, Tokyo, Japan) at a resolution of 7 keV.

### 3.6. Antibacterial Test

In this experiment, the antibacterial activities of Ag NPs synthesized using the PAAc nanogels against *S. aureus* ATCC 6538P and *E. coli* ATCC 25922 have been studied on solid growth media. The freshly grown bacterial inoculums (10^6^ cell/mL, 100 μL) were prepared and seeded on a Petri dish (87 × 15 mm) containing nutrient agar (NA) and pre-incubated in a 37 °C oven for 3 h. The plates were then supplemented with PAAc nanogels (6 mm diameter, intercepted by biopsy punch, Seki, Japan) and incubated at 37 °C for 24 h until a growth inhibition zone could be seen.

### 3.7. *In Vivo* Wound Healing

Three experimental groups, namely, (a) non-treated control wound, (b) the PAAc nanogel paste, and (c) Ag/PAAc nanogel paste, were tested for *in vivo* wound healing.

The female db/db mice (C57BLKS/J Iar- + Lepr^db^/+Lepr^db^, weighing between 18 and 24 g, aged 5 weeks) were used for *in vivo* wound healing assessment. The nanogel pastes were prepared by mixing with distilled water after freeze drying, used for wound dressing and *in vivo* wound healing compared to an untreated control. The skin of the mouse was shaved and disinfected using 70% ethanol. Two full thickness skin wounds were prepared by a skin compressor for 24 h on the dorsum of each mouse’s body after acclimatization for one week. The wound dressing was changed every 2 days, and the rate of wound contraction and microscopic observations were assessed for two weeks. Real-time photograph observations were followed, and the healing rates of the wound area contractions were calculated after 1, 3, 7, 10, 14 and 21 days. Three experimental groups for ulcer care were tested five times, respectively.

## 4. Conclusions

In this study, we have prepared Ag/PAAc nanogel in a one step process by using electron beam irradiation. We confirmed that Ag/PAAc nanogels were synthesized at about a 200 nm scale and were spherical in shape without any catalyst by the electron-beam irradiation. The size of the Ag/PAAc nanogels decreased with increasing irradiation doses, and the absolute value of the zeta potential increased with increasing irradiation doses. Also, the Ag/PAAc nanogels exhibited good antibacterial activity against both Gram-negative and Gram-positive bacteria. In the results of the *in vivo* wound healing, the Ag/PAAc nanogels have a good healing effect. In conclusion, the Ag/PAAc nanogels prepared by electron-beam irradiation may contribute to their application as biomaterials in drug delivery systems.

## Figures and Tables

**Table 1:** Antibacterial effect data of Ag/PAAc nanogel against *Escherichia coli* and *Staphylococcus aureus*; PAAc 1 wt%, hexane 3 wt% and AgNO_3_ 0.005 M.

Radiation dose (kGy)	Gram Negative	Gram Positive

	*E. coli*	*S. aureus*

	Inhibition zone (mm)	
0	0.4 ± 0.1	1.1 ± 0.2
10	0.5 ± 0.1	0.7 ± 0.2
30	1.1 ± 0.3	1.3 ± 0.2
50	1.5 ± 0.4	1.6 ± 0.2
75	2.2 ± 0.6	2.6 ± 0.4
100	3.7 ± 0.5	3.5 ± 1.2
150	4.4 ± 1.0	3.7 ± 1.3
